# Development and Feasibility Testing of a Video Game to Reduce High-Risk Heterosexual Behavior in Spanish-Speaking Latinx Adolescents: Mixed Methods Study

**DOI:** 10.2196/17295

**Published:** 2020-05-04

**Authors:** Meredith J Pensak, Lisbet S Lundsberg, Nancy L Stanwood, Abigail S Cutler, Aileen M Gariepy

**Affiliations:** 1 Department of Obstetrics and Gynecology University of Cincinnati College of Medicine Cincinnati, OH United States; 2 Department of Obstetrics, Gynecology and Reproductive Sciences Yale School of Medicine New Haven, CT United States

**Keywords:** adolescent, Latino, Latina, sexual activity, video game

## Abstract

**Background:**

Similar to broader health disparities, Latinx adolescents have higher rates of high-risk sexual behavior resulting in pregnancy rates that are 2 times higher and sexually transmitted infection rates that are 5 to 8 times higher than non-Hispanic, white adolescents. Novel approaches are needed to reduce high-risk sexual behavior among Spanish-speaking Latinx adolescents who represent the fastest-growing group of US immigrants.

**Objective:**

This study aimed to partner with Spanish-speaking Latinx adolescents in a participatory design process to develop and test a Spanish-language video game intervention to decrease high-risk heterosexual behavior.

**Methods:**

This is an iterative, two-phase, mixed methods study. In phase 1, we conducted focus groups with Spanish-speaking Latinx adolescents to elicit feedback on the content and format of an existing English-language video game. Feedback was then incorporated into an expanded and culturally adapted Spanish-language video game. In phase 2, we pilot tested the feasibility, acceptability, and preliminary efficacy of the new Spanish-language video game intervention by measuring known antecedents to sexual behavior (intentions, self-efficacy, risk perception, and knowledge) assessed at enrollment and 12-week follow-up. We applied a thematic analysis to examine focus group feedback and a bivariate analysis to analyze pre- and postquantitative data.

**Results:**

In phase 1, 15 Spanish-speaking Latinx adolescents provided feedback for further video game development. A Spanish-language video game was then produced and tested in phase 2. We recruited and enrolled 24 Spanish-speaking Latinx adolescents aged 15 to 17 years. Participants played the video game for an average of 4.2 hours during monitored sessions. Pilot testing demonstrated feasibility and acceptability; 65% (3/20) of participants stated that they would play it again, and 65% (3/20) said they would recommend it to friends. Condom-specific knowledge did significantly increase between baseline and follow-up (*P*=.007). Other variables of sexual behavior antecedents did not differ significantly between baseline and 12-week follow-up.

**Conclusions:**

An iterative participatory design process in partnership with Spanish-speaking adolescents produced an innovative and acceptable Spanish-language video game intervention aimed at decreasing high-risk sexual behavior in adolescents. Pilot testing demonstrated preliminary feasibility and yielded essential information for further video game development.

## Introduction

### Background

Adolescents are susceptible to high-risk sexual behaviors, such as having vaginal intercourse without a condom, multiple sexual partners, and intercourse under the influence of drugs and alcohol. Such behaviors expose individuals to an increased risk of sexually transmitted infections (STIs) and unintended pregnancy [1]. However, not all adolescents are at the same risk for these behaviors and consequences. Health disparities account for marked variations in rates of STIs and unintended pregnancy among different subgroups of teenagers. Latinx adolescents are a high-risk population for STIs and unintended pregnancy. Chlamydia rates for Latina adolescents are 17.8 per 1000 adolescents compared with 14.3 per 1000 white female adolescents [2]. Latino adolescents also have higher rates of chlamydia compared with white male adolescents, 41.4 versus 26.6 per 1000, respectively [2]. Although the pregnancy rate among Latina adolescents is lower than the pregnancy rate in non-Latina black adolescents, it is significantly higher than the pregnancy rate in non-Latina white adolescents; there are 61 pregnancies per 1000 Latina teens, 76 pregnancies per 1000 black teens, and 30 pregnancies per 1000 non-Latina white teens [3].

Understanding the experiences of the US Latinx adolescent community is vital, given that Latinx adolescents currently contribute to the fastest-growing group of immigrants in the United States [4]. According to the 2010 US Census, Hispanics comprise 16.3% of the population [4]. Of note, the terms *Latino* and *Hispanic* are often used interchangeably. The US Census defines “Hispanic or Latino as a person of Cuban, Mexican, Puerto Rican, South or Central American, or other Spanish culture or origin regardless of race” [5]. Neither of these terms include the current language spoken, and over 50% of Hispanic adults do not have a preference for being identified as either Latino or Hispanic [6]. In this paper, we used Latino to refer to our target population. For references to population data, such as the US Census, we used the term that was used in the survey. Between 2000 and 2010, the Hispanic population grew by 43%, of which 23% are aged 17 years or younger—a 39% increase over the last decade [4]. Millennial age and younger Latinx adolescents are increasingly more proficient in English; however, 90% still speak Spanish as their primary language at home [7]. Given these demographic changes, it is increasingly important to consider language preference and cultural context when designing health interventions for Latinx adolescents.

Existing public health efforts to decrease high-risk sexual behavior among adolescents are informed by an epidemiologic and cultural understanding of risk factors. Conventional interventions are often delivered within school-based curricula or self-directed learning modules. Recently, there has been a pressing need to diversify approaches aimed at decreasing adolescent high-risk sexual behavior and a call for creative solutions [8,9]. Technology in the form of video games provides an ideal avenue to reach teens: according to the Centers for Disease Control and Prevention (CDC) Youth Risk Behavior Surveillance survey, 41.7% of high school students reported playing a video game, computer game, or using the computer for nonschool purposes for 3 or more hours per day [10]. About 50% of youths reported playing video games daily [11]. The emerging science of serious games, defined as video games for purposes other than just entertainment, holds a special potential to promote healthy behaviors among teenagers [11,12]. Through interactive simulations, serious games offer adolescents the opportunity to practice decision making (eg, whether to use a condom) and experience consequences (eg, positive pregnancy test) in safe, virtual learning environments. A recent randomized controlled trial in young (mean age 12.9 years), minority adolescents showed that an interactive serious video game led to improved sexual knowledge and sexual health attitudes [13]. Similarly, a pilot study to create and test an English-language video game among black and Latinx adolescents to target known antecedents of high-risk sexual behavior showed improvements in sexual knowledge, self-efficacy, and risk perception [14]. Latinx participants in the English-language pilot study suggested developing an expanded and culturally adapted Spanish-language video game to meet the needs of the growing Spanish-speaking Latinx population in the United States [14], which formed the basis for this study.

### Objectives

We designed a 2-phase pilot study in direct partnership with Spanish-speaking Latinx adolescents. In phase 1, we conducted focus groups with Spanish-speaking Latinx adolescents to elicit feedback on the existing English-language video game [14] and employed a participatory design to develop an expanded and adapted Spanish-language video game aimed at addressing high-risk heterosexual behavior to include content that was culturally relevant and responsive to Latinx teens. In phase 2, we pilot tested the feasibility and acceptability of the new Spanish-language video game intervention among Spanish-speaking Latinx adolescents aged 15 to 17 years. We also evaluated the video game’s preliminary efficacy by examining antecedents to and actual high-risk sexual behaviors among Spanish-speaking Latinx adolescents. This paper aimed to describe the development and feasibility testing of this video game to reduce high-risk heterosexual behavior in Spanish-speaking Latinx adolescents.

## Methods

### Study Design

**Figure 1:**
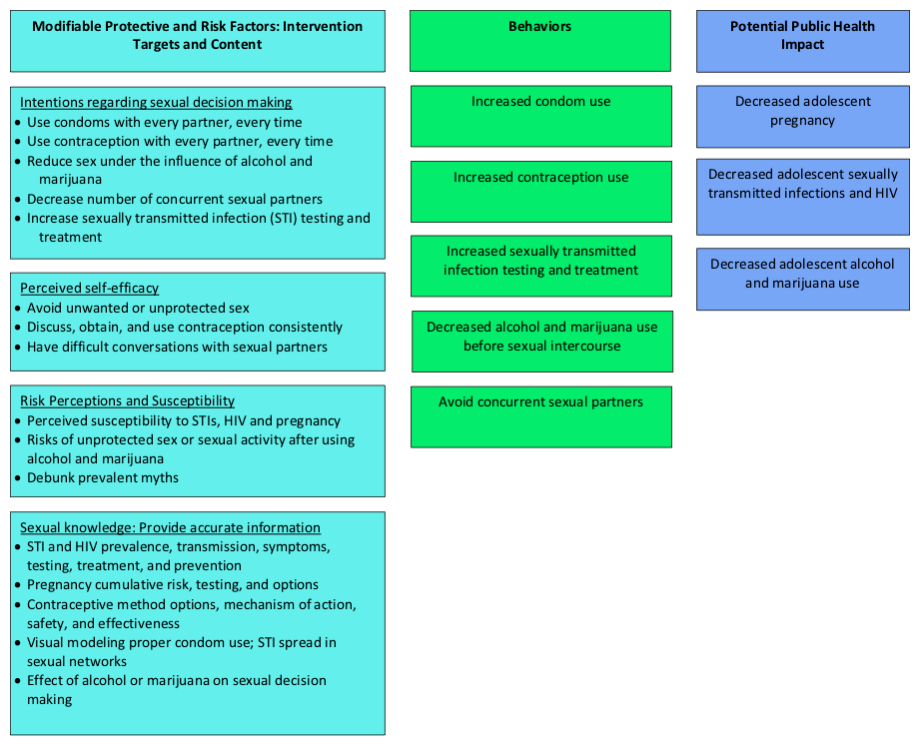
Focused logic model.

We conducted a pilot study with a mixed methods design that built on an existing English-language video game prototype utilizing a theory-driven framework (Figure 1) to target known antecedents of adolescent sexual behavior. This framework [15-17] triangulates strategic components of 3 theoretical models to effect behavior change by targeting modifiable protective and risk factors associated with adolescent sexual risk-taking behavior, including improving intentions regarding sexual decision making, perceived self-efficacy, risk perception and susceptibility, and sexual knowledge [15-38]. The Theory of Planned Behavior posits that an individual’s *intention* to engage in a specific behavior predicts that behavior. The Health Belief Model stresses the importance of individuals’ *self-efficacy* to accomplish a given behavior, *perceptions of susceptibility* to a given health problem, and *knowledge* of and beliefs in the effectiveness of an intervention. The Social Cognitive Theory proposes that knowledge is insufficient to change behavior and emphasizes that observational learning, such as seeing modeled behavior and its consequences or, more importantly, providing individuals with the opportunity to practice skills (as can occur in video game simulation), may enhance behavioral change. An adolescent brain development theory, namely emotional and social self-efficacy [25] and impulsivity (sensation seeking, negative urgency, positive urgency, lack of premeditation, and lack of perseverance) [30], was also incorporated into our design. This theoretical foundation and its key intervention targets, which have been shown to improve black and Latinx adolescent sexual risk-taking behavior (eg, increased STI testing and treatment) [17-38], informed the video game intervention framework and content (Textbox 1).

The existing English-language video game previously was developed and tested with black or Latinx adolescents aged 15 to 17 years, who suggested that the video game setting be a party scene populated by avatars (ie, a digital representation of a person playing the game) [14]. The player then makes decisions for multiple avatars (eg, the Hand of God viewpoint) that simulates decision making in real-life scenarios regarding alcohol or marijuana use, sexual activity, and use of condoms and/or other contraception. Finally, the video game modeled short- and long-term consequences of decisions regarding sexual behavior, such as STI acquisition and pregnancy [14].

This study was conducted in 2 phases: a formative phase (phase 1) and a testing phase (phase 2). During phase 1, we conducted focus groups with Spanish-speaking (English-proficient) Latinx adolescents to modify and shape the existing English-language video game with maximum input from our target audience. Then the video game was adapted and modified in response to participants’ feedback and suggestions. In phase 2, we evaluated the following aspects of feasibility and acceptability of the gameplay experience: usability, engagement, satisfaction, game completion, and preliminary efficacy.

**Description of framework and content of Colegio del Sexperto (Sexpert High School), a video game intervention to reduce high-risk sexual behavior in Spanish-speaking Latinx adolescents.**

- Type of video game: role-playing, simulation, educational
- Player’s objective: Become a sex expert (sexpert). Unlock information and answer trivia to gain points, accumulate avatars, and reach higher video game levels
- Video game design:
  - *Party*
    1. Game starts at a party where player controls multiple avatars (digital representations of partygoers), conversing, making decisions
    2. Player guided to unlock key information about sex and answer trivia questions, both of which earn points
    3. Player makes decisions for avatars regarding alcohol/marijuana use, sex, using a condom and/or effective contraception
    4. If player uses alcohol or marijuana, a condom and/or effective contraception may not be available
  - *Party results*
    1. For every avatar that was sexually active, player spins a wheel to view the consequences of decisions, including possible pregnancy (based on the use of alcohol, marijuana, condom, or other contraception use)
    2. Player is presented with a choice to go to a clinic for sexually transmitted infection (STI) testing and/or to discuss and choose contraception
    3. After receiving test results, the player is led to a conversation with a sexual partner to discuss results and any concerns
  - *Advancement*
    1. Advance to next level, gain more avatars, have options to get STI testing, and obtain condoms and/or contraception before the next party
    2. Presented with new scenarios and choices at the next level, as mentioned earlier
- Video game content: Known antecedents to sexual behavior including intentions, self-efficacy, risk perceptions and susceptibility, and knowledge
- Components targeting behavior change:
  - Skill building via simulations of difficult discussions about sex and decision making in scenarios like real life
  - Modeling short- and long-term consequences of decisions (eg, contraception use and STI testing and treatment)
  - Incentivization by earning points within the video game
- Transferrable skills:
  - Communication: discuss whether to have sex, sex refusal, contraception negotiation, and HIV/STIs and pregnancy testing and results
  - Evaluating risk: how the use of alcohol and marijuana can influence sexual risk taking
  - Decision making: communication, take pregnancy tests, STI testing and treatment, and condoms and contraception
  - Problem solving: where and how to obtain pregnancy tests, STI testing and treatment, and condoms and contraception

### Recruitment

We partnered with a racially, ethnically, and socioeconomically diverse public high school in New Haven, Connecticut. Participants were recruited through flyers at the school and during presentations in English as a second language classes. Enrollment criteria for both phases included female or male adolescents who identified as Latino or Hispanic, aged 15 to 17 years, fluent in Spanish, and proficient in English. There were no enrollment criteria related to prior sexual experience. In phase 1, only nonidentifying demographic information was collected. Parents were notified of their adolescents’ desire to participate in the study and were given the chance to decline participation on behalf of their children. Verbal assent from adolescents was required for participation. In phase 2, potentially identifying demographic information was collected, and both parental and adolescent written consent was required for participation. The study protocol was approved by the Yale University Human Investigation Committee and was granted a certificate of confidentiality from the Department of Health and Human Services to guarantee the protection of participants’ privacy.

### Formative Phase—Phase 1

Bilingual moderators trained in conducting focus groups facilitated all phase 1 focus group sessions, which lasted approximately 75 min each. Focus groups were conducted separately by those of self-identified same gender to maximize comfort, minimize self-consciousness, and encourage open discussion [39]. First, participants spent 30 min playing the English version of the video game on individual tablets; this was followed by a group discussion until thematic saturation was reached across multiple focus groups. We used a semistructured interview guide comprising open-ended questions to lead the discussion. Questions explored adolescents’ attitudes and perceived social norms regarding drug and alcohol use, sexuality, monogamy, fidelity, pregnancy, contraception, and abortion, and also attitudes toward and experiences with romantic relationships, decision making with respect to sex initiation, drug and alcohol use separate from or concomitant with sexual encounters, use of contraception and/or STI protection, and the issue of who has control in romantic relationships and why [1,19,34]. We also assessed participants’ interest in playing a video game designed to improve knowledge about the implications of sexual behavior and drug use and to simulate and practice decision making in a virtual setting and explored culturally relevant content and video game features that would be responsive to Latinx teens. We audio-recorded interviews that were translated and transcribed by native Spanish speakers. We analyzed transcripts using a thematic analysis to identify key themes and specific domains of impact [40,41].

### Testing Phase—Phase 2

Adolescents participating in phase 2 played the video game weekly at monitored 1-hour sessions for a total of 6 weeks. Video gameplay was done individually on personal tablets. Participants were encouraged not to talk or interact during video gameplay time and were monitored to ensure individual, independent gameplay. Tablet devices were numbered, and participants were given the same tablet each time they played. Self-administered, paper survey assessments were given at 3 time points: upon phase 2 enrollment, immediately after the last gameplay session, and at 12-week follow-up. We collected baseline demographic information at the time of enrollment. Given the short time frame of the study period and small sample size of the pilot study, preliminary efficacy of the video game was assessed with variables known to be antecedents of high-risk sexual behavior including intentions, self-efficacy, risk perceptions, and sexual knowledge. These were assessed at the time of phase 2 enrollment, immediately after the last gameplay session, and at 12-week follow-up. We also collected data on actual sexual behaviors such as frequency of intercourse, condom use, contraception use, number of partners, intercourse under the use of drugs and alcohol, and diagnoses of STIs and pregnancy at enrollment and follow-up. Survey questions came from validated tools, including the CDC Youth Risk Behavior Surveillance, the safer sex intention scale, and self-efficacy for negotiating condom use [42-44].

Video game acceptability and feasibility were assessed with qualitative and quantitative data. Qualitative feedback was collected during focus groups after the completion of gameplay. A total of 4 focus groups were conducted by the same bilingual moderators as in phase 1 and were audio-recorded, translated, transcribed, and analyzed using a thematic analysis to identify key concepts. Quantitative data on gameplay acceptability and feasibility, including how long each participant played for, maximum game level reached, and the number of sessions attended were collected as part of the survey following the completion of gameplay. Additional quantitative survey data were collected following the completion of gameplay and 12 weeks after exposure to the video game to assess video game visual acceptability, like/dislike of the video game, connection to the avatars, frustrations with the video game, and desire to play the video game again or recommend to friends.

### Statistical Analysis

We assessed demographics and participant characteristics using descriptive statistics. The preliminary efficacy of the video game was assessed by examining mean summary scores for intentions, self-efficacy, risk perception, and knowledge. Individual questions were measured using 5-point Likert scales where 4=I strongly agree, 3=I agree, 2=I am not sure, 1=I disagree, and 0=I strongly disagree. The statements were worded in the affirmative. For example, “If I have sex without contraception (something to prevent pregnancy), I would probably get pregnant (or get someone pregnant).” Higher scores indicated an improvement in the respective categories; 1 question was reverse coded. Mean summary scores were calculated for the variables measuring the preliminary efficacy of the video game within the following categories: intentions, self-efficacy, risk perception, and knowledge. Mean summary scores for intention (0-64), self-efficacy (0-44), and risk perception (0-44) were cumulative, based on Likert values 0 to 4 for individual questions. Mean knowledge scores (0%-100%) were calculated as the percentage correct. We calculated the Cronbach α for each scale to examine internal consistency. Cronbach α scores ranged from .70 to .80, demonstrating good overall reliability. Knowledge questions were asked with *true/false/not sure* answers, and the total percentage correct was reported. Baseline and 12-week follow-up assessment scores were compared with a Student *t* test, and *P* values were calculated.

## Results

### Formative Phase—Phase 1

A total of 15 Spanish-speaking Latinx adolescents participated in 4 phase 1 focus groups, each consisting of 4 to 6 participants. Out of 15 participants, 7 (47%) were female, and 12 (80%) participants were born outside of the continental US. After 30 min of gameplay, participants overwhelmingly liked the premise of the video game, including the process of simulated decision making using avatars, virtually experiencing consequences of those decisions, getting feedback on the decisions, having opportunities for *do-overs*, and the chance to answer trivia questions, which allowed them to test their knowledge and gain new information. Participants recommended modifying the existing video game, as opposed to developing a new game. Key suggestions to generally improve the English-language video game included adding content to increase gameplay time, decreasing the length and overall amount of text, adding more animation to avatars, and increasing the number of video game background settings (eg, party and bedroom). One major Latinx-specific theme that emerged and that teens recommended to be incorporated into the video game was the importance of involving family members and close friends in the decision-making process around dating, sex, contraception, and pregnancy. This underlines the importance of *familismo*, the loyalty that Latinx individuals often feel toward extended family over personal needs that is present in Latinx culture [44]. Participants suggested modeling difficult conversations with parents in the video game to meet their needs of family engagement in their decision-making processes; participants wanted to simulate both positive and negative conversations to have these skills to use in their real lives. Additional Latinx-specific themes that emerged included the need to address perceptions of *fatalismo*, the belief that individuals cannot control when or if pregnancy occurs (even if contraception is used) because it is part of their destiny regarding pregnancy [45], and the incorporation of more opportunities to navigate decisions regarding long-term (novio and novia) and casual relationships. These themes were incorporated into the next iteration of the video game prototype, which was also translated into Spanish by bilingual speakers, including native Spanish speakers.

### Testing Phase—Phase 2

#### The Intervention

We named our Spanish-language video game *Colegio del Sexperto*, which means Sexpert High School. The goal of the 12-level video game is to become a sex expert (sexpert), which players do by working through multiple simulated scenarios to practice decision making around sexual behavior and experience consequences of those decisions, all while accumulating knowledge (Textbox 1). We identified 9 thematic learning objectives of the video game that decision-making scenarios and trivia questions incorporated: spread of STIs in sexual networks; STIs and HIV risk, testing, and treatment; cumulative risk of pregnancy with unprotected sex; contraception; possible consequences of pregnancy; effects of alcohol and drug use; partner communication regarding STIs; partner communication regarding contraception; and partner communication regarding pregnancy [18,19]. The learning objectives were spread out across 12 levels to facilitate players' decision-making simulation and knowledge accumulation in a step-wise fashion with the goal of not having players stay on each level for too long. Players *win* the video game when they complete all 12 levels.

**Figure 2:**
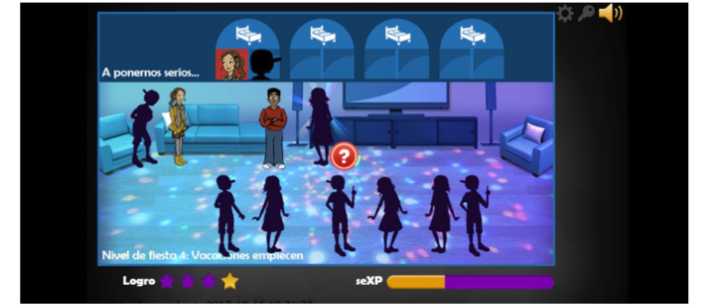
Screenshot from Colegio del Sexperto (Sexpert High School) video game illustrating avatars interacting and trivia questions.

Through a *Hand of God* view, players guide multiple male and female avatars through a house party (Figure 2). Each level begins with a new party and an additional avatar. At the party, the player-guided avatar interacts with computer-guided avatars. The computer-guided avatars offer alcohol, marijuana, and the opportunity to *hook-up*. Player-guided avatars can advance from the dance floor at the party to the bedroom for the *hook-up*, while making decisions for their avatars about whether to use alcohol or marijuana, engage in sexual activity at a party, and use condoms and/or contraception. Players then follow their avatars as they experience the outcomes (eg, pregnancy and STIs) of those decisions. Upon completion of each level, players have simulated conversations in which they disclose STI and/or pregnancy test results to sexual partners, friends, and family members. They are given opportunities to seek (or decline) treatment for STIs and to obtain condoms and other forms of contraception before starting the next party.

Player-guided avatars also answer trivia questions that address the main thematic learning objectives of the video game (eg, risks of STIs, effects of drugs and alcohol on sexual behavior, and partner communication). Players gain points by answering trivia questions correctly, and a minimum of trivia questions must be answered correctly to obtain enough points to advance to the next level.

#### Participant Characteristics

In phase 2, we recruited and enrolled 24 Spanish-speaking Latinx adolescents. The average age of participants was 15.8 years (range 15-17), and there were equal numbers of male and female participants (Table 1). Out of 24 participants, 14 (59%) were born in Puerto Rico; the remaining reported being born outside of the United States. Out of 23 participants, 14 (61%) reported living in continental United States for 1 to 5 years, 87% (21/24) participants reported speaking only or mostly Spanish at home, and 58% (14/24) participants reported speaking only or mostly Spanish with friends. At baseline, 7 out of 24 (29%) participants reported any prior sexual experience, and within this group, all reported using only condoms or no method of contraception at the time of last intercourse. Only 1 participant reported the use of drugs or alcohol at the time of last intercourse (Table 1).

**Table 1:** Phase 2 baseline characteristics (N=24).

Characteristic		Values
Age (years), mean (range)		15.8 (15-17)
**Sex/gender, n (%)**		
	Female	12 (50)
	Male	12 (50)
**Race, n (%)**		
	American Indian or Native Alaskan	4 (19)
	Black or African American	0 (0)
	Native Hawaiian or other Pacific Islander	1 (5)
	White	3 (14)
	Other	13 (62)
**Country of birth, n (%)**		
	United States	0 (0)
	Mexico	2 (8)
	Puerto Rico	14 (58)
	Dominican Republic	4 (17)
	Other	4 (17)
**Time in the United States (years), n (%)**		
	<1	7 (30)
	1-5	14 (61)
	5-10	2 (9)
**Language spoken at home, n (%)**		
	Only or mostly Spanish	21 (86)
	Spanish and English	3 (13)
	Only or mostly English	0 (0)
**Language spoken with friends, n (%)**		
	Only or mostly Spanish	14 (58)
	Spanish and English	9 (38)
	Only or mostly English	1 (4)
**Self-reported behaviors at baseline^a^**		
	Ever had sexual intercourse, n (%)	7 (29)
	Mean age of first intercourse (SD)^b^	14.6 (1)
	≥2 lifetime sexual partners^b^, n (%)	3 (43)
**Contraception used at last sexual intercourse^a^, n (%)**		
	Only condoms^b^	5 (71)
	No method^b^	2 (29)
Use of alcohol or drugs at last sexual intercourse^a^, n (%)		1 (14)

^a^On the basis of questions used in the Centers for Disease Control and Prevention, Youth Risk Behavior Surveillance.

^b^Percentage out of only sexually active participants (n=7).

#### Gameplay Experience

On average, participants played 4.2 hours of the video game over the 6-week time period. Out of 24 participants, 14 (58%) reached level 5, and 2 (8%) participants successfully completed all 12 levels of the video game. At the 12-week follow-up, 65% (13/20) participants reported that they enjoyed playing the video game, would play it again, and would tell their friends to play the video game (see the table in Multimedia Appendix 1). Out of 20 participants, 16 (80%) participants felt responsible for the choices they made in the video game, 15 (75%) participants liked the way it looked, and 14 (70%) participants found the video game challenging; 8 (40%) participants felt frustrated playing the video game. When asked to elaborate on these frustrations in focus groups, participants reported that the video game was repetitive, had too many levels, and was unnecessarily challenging (eg, losing avatars without knowing why or getting stuck on a given level). Nevertheless, they reported that they liked playing the video game and described it as an improvement over school-based sex education classes. Suggestions for future iterations of the video game included adding more avatars, diversifying the information encountered in each level, providing in-game clues for how to advance to the next level, condensing the number of levels, and further improving animation of the avatars.

#### Impact of the Video Game on Intentions, Self-Efficacy, Risk Perception, Knowledge, and Sexual Behavior

Mean summary scores for intentions, self-efficacy, risk perception, and knowledge did not change significantly between baseline and 12-week follow-up assessments (Table 2). However, mean summary knowledge scores did increase from 27% correct to 33% correct (*P=*.12). On the basis of the finding that most players (14/24, 58%) did not get past level 5 of the video game, we explored how content was distributed throughout the video game. An in-depth review revealed that condom-specific information, although a central focus of the video game overall, was heavily represented in the first 3 levels of the video game, and 92% (22/24) participants played through the levels in which condom-specific information was addressed. Therefore, we performed a subanalysis of condom-specific questions. The mean summary scores for condom-specific measures were compared between baseline and 12-week follow-up. Condom-specific knowledge scores significantly increased from 29.6% to 47.4% correct (*P*=.007, Table 3). Condom-specific intentions, self-efficacy, and risk perception did not significantly change. At the 12-week follow-up survey, 45% (9/20) of participants reported being sexually active compared with 29% (7/29) at enrollment; however, this was not significant, and data were not shown here. Of the sexually active participants at 12-week follow-up, 80% (4/5) reported condom use at the time of last intercourse compared with 71% (5/7) at baseline, again not a significant difference. Among those individuals who were sexually active at the 12-week follow-up (n=7), all reported not using alcohol or drugs at the time of last intercourse.

**Table 2:** Participant summary scores for key intervention targets before video game intervention and at 12-week follow-up.

Mean outcome summary scores^a^	Baseline, mean (SD)	12-week follow-up, mean (SD)	*P* value (baseline to follow-up)
Intention score (0-64)^b^	42.68 (8.03)	42.95 (7.94)	.75
Self-efficacy score (0-44)^c^	31.39 (5.57)	32.55 (7.18)	.42
Risk (misperception) score (0-44)^d^	29.38 (5.28)	26.65 (6.62)	.14
Knowledge score (0%-100%)^e^	26.71 (11.25)	32.77 (14.82)	.12

^a^Higher scores indicate improved intentions, self-efficacy, and risk perceptions as measured using a 5-point Likert scale where 4=I strongly agree, 3=I agree, 2=I am not sure, 1=I disagree, and 0=I strongly disagree. The statements are worded in the affirmative. For example, “If I have sex without contraception (something to prevent pregnancy), I would probably get pregnant (or get someone pregnant).”

^b^16 questions; Cronbach α=.80; n=18 [19,35].

^c^11 questions; Cronbach α=.73; n=19 [19,46,47].

^d^11 questions; Cronbach α=.70; n=19 [18,19].

^e^35 questions; percent correct out of 35 questions; n=14 [1,35,48].

**Table 3:** Condom-specific questions—summary scores before and at 12-week follow-up.

Mean outcome summary scores^a^	Baseline, mean (SD)	12-week follow-up, mean (SD)	*P* value baseline to follow-up
Intention score (0-16)^b^	11.96 (2.26)	11.50 (3.46)	.73
Self-efficacy score (0-36)^c^	25.96 (4.93)	26.80 (5.85)	.5
Risk perception score (0-4)^d^	2.04 (1.27)	2.00 (1.84)	.72
Summary knowledge score (0%-100%)^e^	29.55 (19.11)	47.37 (22.27)	.007^f^

^a^Higher scores indicate improved intentions, self-efficacy, and risk perceptions as measured using a 5-point Likert scale where 4=I strongly agree, 3=I agree, 2=I am not sure, 1=I disagree, and 0=I strongly disagree. The statements are worded in the affirmative. For example, “If I have sex without contraception (something to prevent pregnancy), I would probably get pregnant (or get someone pregnant).”

^b^4 questions (n=18).

^c^9 questions (n=19).

^d^2 questions (n=19).

^e^8 questions, percent correct out of 8 questions (n=14).

^f^Statistically significant result.

## Discussion

### Principal Findings

We successfully partnered with Spanish-speaking Latinx adolescents to further develop and adapt an existing English-language video game intervention designed to decrease high-risk heterosexual behavior and adapted it to meet the specific needs of Spanish-speaking Latinx adolescents. Following a participatory, iterative video game design and development process with our target audience, our pilot testing of *Colegio del Sexperto* exhibited feasibility, including the production of a useable Spanish-language video game that included culturally relevant and responsive content. This pilot testing also demonstrated high acceptability: study participants liked the video game and reported feeling invested in the decisions they made during gameplay. Pilot testing also produced thoughtful feedback and detailed suggestions for ways to improve future iterations of the video game, including improving avatar animation, adding more avatars and content, condensing the number of levels, and improving game flow. Our pilot study meets Bowen et al [49] proposed criteria for a successful feasibility study.

We observed improved overall knowledge and significantly increased condom-specific knowledge after exposure to the video game. Although we did not find a significant improvement in intentions, self-efficacy, and risk perception, a larger sample size specifically powered to detect such differences might reveal them. In addition, only 29% (7/24) of participants reported any previous sexual experience at baseline (compared with 48.6% of Hispanic high school students nationwide) [1]. Testing intentions, self-efficacy, and risk perception among a group of sexually active adolescents might yield different results.

Our findings support a growing body of evidence showing that serious games or video games designed for health-related topics have the potential to influence sexual health and behaviors, [50,51] and that there is a need for more culturally specific games to increase engagement and efficacy of such interventions [52]. Video games and mobile health interventions have the ability to cross language and cultural barriers [50,53,54], which makes them especially useful for reaching Spanish-speaking Latinx adolescent populations. The video game developed in this pilot study met adolescents where they are today—playing video games. Moreover, our work adds to the body of emerging research, demonstrating that serious games can be used for purposes other than entertainment [50-52]. Additionally, video games are multi-component and can incorporate strategies to improve modifiable behaviors. This modality can capitalize on experiential learning by offering opportunities to demonstrate social norms, practice self-efficacy skills, visually model risk and risk prevention, and allow adolescents to experiment with and experience consequences of high-risk behaviors in a risk-free virtual world [11,13,14,51,55-58].

### Limitations

As is the nature of a pilot study, our study is limited by its small sample size, lack of control group, and relatively short follow-up interval. Recruitment criteria did not include prior sexual experience; this allowed for a larger recruitment pool but potentially limited our ability to reach an audience that might benefit most from a video game–based intervention. Although our study did include a certificate of confidentiality to guarantee the protection of privacy, the contemporary political climate’s anti-immigrant rhetoric may have contributed to distrust among our target audience of Spanish-speaking Latinx adolescents, which likely hindered the study recruitment and participant retention. Teachers who assisted with participant recruitment noted that some otherwise eligible adolescents expressed hesitancy in joining a research study because they or their family members were undocumented immigrants. Similarly, we had difficulty maintaining ongoing contact with our participants because of the reluctance to share contact information and conflicts between our testing period and the school holiday schedule. As a result, only a small number of participants ultimately completed the immediate postgameplay survey, which limited our ability to compare scores between time points and our analysis of preliminary efficacy. Consistent assurance of anonymity is crucial as are alternative ways of contacting participants (such as adolescent-friendly apps, such as Snapchat or Twitter). Future studies should take special care to create strong partnerships with community members to garner trust and acceptance, especially when working with vulnerable populations. Finally, opportunities exist to improve the mechanics of the video game to decrease the number of levels and make it less challenging, which could increase exposure to the game content. Larger clinical trials are needed to test the intervention against a control group before promoting this intervention on a larger scale.

### Conclusions

Through an iterative intervention design in partnership with our target audience, we developed an innovative and usable Spanish-language video game aimed at decreasing high-risk heterosexual behavior in Latinx adolescents. Findings from this pilot study support the growing body of evidence that serious video games may be used to both increase knowledge and modify behaviors. Our study addresses current gaps in research on culturally sensitive games for health [52]. Pilot testing yielded valuable information and feedback that will aid in future video game development. The next steps include incorporating participants’ specific suggestions into the next video game iteration and testing it in larger studies to adequately examine whether the intervention can decrease high-risk heterosexual behaviors among Latinx adolescents.

## Appendix

Multimedia Appendix 1 Reports of gameplay experience assessed at 12-week follow-up (N=20).

## Abbreviations

CDC: Centers for Disease Control and Prevention
STI: sexually transmitted infection
