# Supplementary material for: Development and Feasibility Testing of a Video Game to Reduce High-Risk Heterosexual Behavior in Spanish-Speaking Latinx Adolescents: Mixed Methods Study
Source: JMIR Serious Games. 2020 May 4;8(2):e17295. doi: 10.2196/17295 (PMC7235807; doi:10.2196/17295)
Supplement: Multimedia Appendix 1 [file games_v8i2e17295_app1.docx]

Table 5. Reports of gameplay experience assessed at 12-week follow-up (N=20)

|  | Strongly Like/Like  N (%) |
| --- | --- |
| I felt responsible for the choices I made in the game. | 16 (80) |
| I like the way the game looked. | 15 (75) |
| The game was challenging. | 14 (70) |
| I would play this game again. | 13 (65) |
| I would tell my friends to play this game. | 13 (65) |
| I enjoyed playing the game. | 13 (65) |
| I would make decisions in life like I made them in the game. | 10 (50) |
| I felt connected to the other characters in the game. | 9 (45) |
| If I were to play this game with my friends, they would influence the way I play. | 9 (45) |
| I felt connected to my character in the game. | 8 (40) |
| If I were to play this game with my friends, I would make different decisions in the game. | 8 (40) |
| I was frustrated with this game. | 8 (40) |
